# (*E*)-2-{[1-Carb­oxy-2-(1*H*-indol-3-yl)ethyl­iminio]meth­yl}-6-hy­droxy­phenolate

**DOI:** 10.1107/S1600536811031709

**Published:** 2011-08-27

**Authors:** Salah Ahmed Ba-Salamah, Naser Eltaher Eltayeb, Siang Guan Teoh, Kong Mun Lo

**Affiliations:** a School of Chemical Sciences, Universiti Sains Malaysia, Minden, Penang, Malaysia; b Department of Chemistry, International University of Africa, Sudan; c Chemistry Department, Faculty of Science, University of Malaya, Malaysia

## Abstract

In the zwitterionic title compound, C_18_H_16_N_2_O_4_, the dihedral angle between the planes of the benzene and indole rings is 26.38 (10)°. An intra­molecular N—H⋯O hydrogen bond generates an *S*(6) ring motif. In the crystal, mol­ecules are linked through N—H⋯O, O—H⋯O and C—H⋯O hydrogen bonds into infinite chains propagating in [010].

## Related literature

For a related structure and background to Schiff bases, see: Ba-Salamah *et al.* (2011 ▶). For other related structures, see: Eltayeb *et al.* (2010*a* ▶,*b* ▶,*c* ▶). For reference bond lengths, see: Allen *et al.* (1987 ▶). For graph-set theory, see: Bernstein *et al.* (1995 ▶).
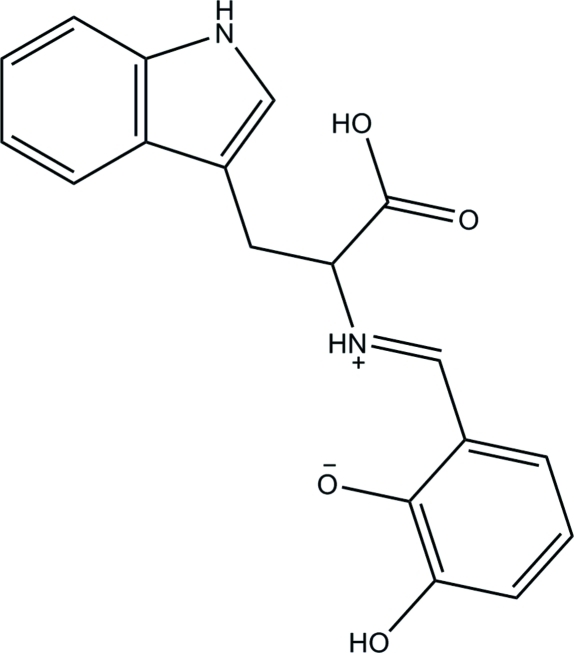


## Experimental

### 

#### Crystal data

C_18_H_16_N_2_O_4_
*M* *_r_* = 324.33
Monoclinic, 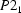

*a* = 8.4351 (2) Å
*b* = 9.3038 (3) Å
*c* = 9.5023 (3) Å
β = 98.683 (2)°
*V* = 737.18 (4) Å^3^
*Z* = 2
Mo *K*α radiation
μ = 0.11 mm^−1^
*T* = 100 K
0.32 × 0.22 × 0.20 mm

#### Data collection

Bruker SMART APEXII CCD diffractometer
Absorption correction: multi-scan (*SADABS*; Bruker, 2009 ▶) *T*_min_ = 0.598, *T*_max_ = 0.746
7473 measured reflections
1949 independent reflections
1781 reflections with *I* > 2σ(*I*)
*R*_int_ = 0.046

#### Refinement

*R*[*F* ^2^ > 2σ(*F* ^2^)] = 0.038
*wR*(*F* ^2^) = 0.093
*S* = 1.14
1949 reflections
233 parameters
1 restraint
H atoms treated by a mixture of independent and constrained refinement
Δρ_max_ = 0.27 e Å^−3^
Δρ_min_ = −0.23 e Å^−3^

### 

Data collection: *APEX2* (Bruker, 2009 ▶); cell refinement: *SAINT* (Bruker, 2009 ▶); data reduction: *SAINT*; program(s) used to solve structure: *SHELXTL* (Sheldrick, 2008 ▶); program(s) used to refine structure: *SHELXTL*; molecular graphics: *SHELXTL*; software used to prepare material for publication: *SHELXTL* and *PLATON* (Spek, 2009 ▶).

## Supplementary Material

Crystal structure: contains datablock(s) I, global. DOI: 10.1107/S1600536811031709/hb6347sup1.cif

Structure factors: contains datablock(s) I. DOI: 10.1107/S1600536811031709/hb6347Isup2.hkl

Additional supplementary materials:  crystallographic information; 3D view; checkCIF report

## Figures and Tables

**Table 1:** Hydrogen-bond geometry (Å, °)

*D*—H⋯*A*	*D*—H	H⋯*A*	*D*⋯*A*	*D*—H⋯*A*
N1—H1*N*1⋯O3	0.97 (3)	1.86 (3)	2.646 (2)	136 (2)
N2—H1*N*2⋯O3^i^	0.90 (3)	2.13 (3)	3.020 (2)	170 (2)
O2—H1*O*2⋯O3^ii^	0.89 (3)	1.64 (4)	2.526 (2)	174 (4)
O4—H1*O*4⋯O3	0.89 (3)	2.45 (3)	2.823 (2)	106 (2)
O4—H1*O*4⋯O1^i^	0.89 (3)	1.83 (3)	2.665 (2)	156 (3)
C7—H7⋯O4^iii^	0.95	2.49	3.197 (3)	132

Symmetry codes: (i) 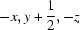; (ii) 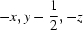; (iii) 
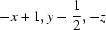
.

**Fig. 1.**
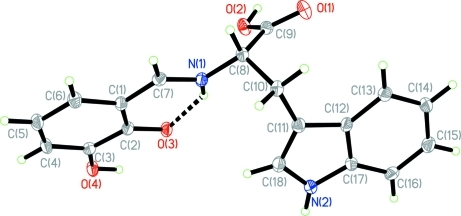
The molecular structure of the title compound, with 50% probability ellipsoids for non-H atoms.

**Fig. 2.**
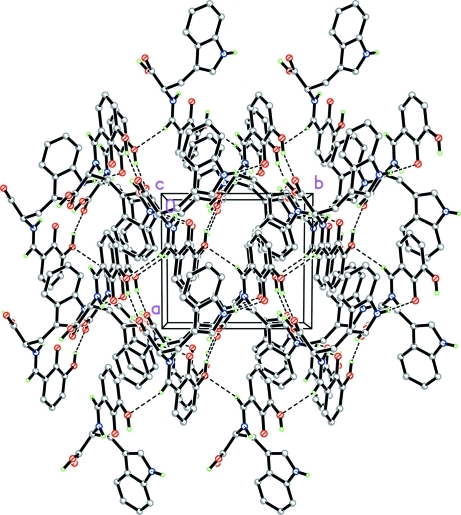
The crystal packing of title compound, viewed down c axis.

## supplementary crystallographic information

### Comment

Recently, we reported the crystal structure of (*E*)-2-(2,4-dihydroxybenzylideneammonio)-3-(1*H*- indol-3-yl)propanoate (Ba-Salamah *et al.* 2011). In this paper, we report the crystal structure of the title compound, (I), (Fig. 1), obtained by the reaction of *L*-tryptophan and 2,3-dihydroxybenzaldehyde.

In the zwitterionic title compound, C_18_H_16_N_2_O_4_, the dihedral angle between the planes of the benzene and indole rings is 26.38 (10)°. Bond lengths have normal values (Allen *et al.*, 1987). The C1 in the benzene ring and C11 in the indol ring are connected together by a chain of four atoms, C7/N1/C8/C10 which has torsion angle 101.2 (2) °. The torsion angles of the chain C8/N1/C7/C1 and N1/C8/C10/C11 are -179.0 (2) ° and 64.4 (2) °, respectively. The title molecule has a zwitterionic form with an intramolecular N1—H1N1···O3 hydrogen bond (Table 1, Fig.1) which generates an S(6) ring motif (Bernstein *et al.*, 1995). The C7—N1 [1.295 (3) Å] and C2—O3[1.318 (3) Å] bond distances are comparable to those [1.3129 (19) and 1.2915 (17) Å; 1.310 (2) and 1.304 (2) Å;1.302 (2) and 1.316 (2) Å] observed in similar zwitterionic structures (Eltayeb *et al.*, 2010*a*,*b*,*c*).

In the crystal structure of (I) as shown in Fig. 2, the molecules are linked through N—H···O, O—H···O and C—H···O hydrogen bonds (Table 1). C—H···π interactions are also present; C8—H8···*Cg*3^i^ = 2.70 Å, C16—H16···*Cg*2^ii^ = 2.81 Å. *Cg*3 and *Cg*2 are centroids of C12—C17 and C1—C6 rings respectively, [symmetry codes: (i) = –*X*,-1/2+Y,1-*Z*; (ii) = –*X*,1/2+Y,-*Z*]. The molecules are linked into infinite one-dimensional chains along the *b* axis.

### Experimental

To a 100-ml round-bottom flask were added 20 ml (3:1) methanol-water and 2 mmol of *L*-tryptophan (0.416 g), the mixture stirred to form a clear colorless solution, 2 mmol of 2,3-dihydroxybenzaldehyde (0.276 g) added to form a yellow clear solution. The mixture was refluxed under heating and stirring for seven hours after which the mixture was filtered and left to stand at room temperature for 12 h. Dark orange crystals which appeared, were filtered after two days and recrystallized from ethanol to yield orange blocks of (I), then left to dry. The IR showed broad peak due to O–H (carboxylic acid and phenol) stretch superimposed on the sharp band due to N–H stretch at 3500–3100 cm^-1^. The C=O stretch (1708 cm^-1^), C=N stretch (1644 cm^-1^), C–O stretch (1230 cm^-1^), O–H bends (1460 cm^-1^).

### Refinement

The N- and O-bound H atoms were located in a difference Fourier map and were refined freely. The remaining H atoms were positioned geometrically and refined using a riding model with C–H = 0.95 or 1.00 Å and *U*_iso_(H) = 1.2*U*_eq_(C). In the absence of significant anomalous scattering effects, 1577 Friedel pairs were merged.

### Crystal data

**Table:**

C_18_H_16_N_2_O_4_	*F*(000) = 340
*M**_r_* = 324.33	*D*_x_ = 1.461 Mg m^−^^3^
Monoclinic, *P*2_1_	Mo *K*α radiation, λ = 0.71073 Å
Hall symbol: P 2yb	Cell parameters from 1920 reflections
*a* = 8.4351 (2) Å	θ = 3.0–27.7°
*b* = 9.3038 (3) Å	µ = 0.11 mm^−^^1^
*c* = 9.5023 (3) Å	*T* = 100 K
β = 98.683 (2)°	Block, orange
*V* = 737.18 (4) Å^3^	0.32 × 0.22 × 0.20 mm
*Z* = 2	

### Data collection

**Table:**

Bruker SMART APEXII CCD diffractometer	1949 independent reflections
Radiation source: fine-focus sealed tube	1781 reflections with *I* > 2σ(*I*)
graphite	*R*_int_ = 0.046
φ and ω scans	θ_max_ = 28.4°, θ_min_ = 2.2°
Absorption correction: multi-scan (*SADABS*; Bruker, 2009)	*h* = −11→11
*T*_min_ = 0.598, *T*_max_ = 0.746	*k* = −12→12
7473 measured reflections	*l* = −12→12

### Refinement

**Table:**

Refinement on *F*^2^	Primary atom site location: structure-invariant direct methods
Least-squares matrix: full	Secondary atom site location: difference Fourier map
*R*[*F*^2^ > 2σ(*F*^2^)] = 0.038	Hydrogen site location: inferred from neighbouring sites
*wR*(*F*^2^) = 0.093	H atoms treated by a mixture of independent and constrained refinement
*S* = 1.14	*w* = 1/[σ^2^(*F*_o_^2^) + (0.0454*P*)^2^ + 0.0637*P*] where *P* = (*F*_o_^2^ + 2*F*_c_^2^)/3
1949 reflections	(Δ/σ)_max_ < 0.001
233 parameters	Δρ_max_ = 0.27 e Å^−^^3^
1 restraint	Δρ_min_ = −0.23 e Å^−^^3^

### Special details

**Table:**

Geometry. All e.s.d.'s (except the e.s.d. in the dihedral angle between two l.s. planes) are estimated using the full covariance matrix. The cell e.s.d.'s are taken into account individually in the estimation of e.s.d.'s in distances, angles and torsion angles; correlations between e.s.d.'s in cell parameters are only used when they are defined by crystal symmetry. An approximate (isotropic) treatment of cell e.s.d.'s is used for estimating e.s.d.'s involving l.s. planes.
Refinement. Refinement of *F*^2^ against ALL reflections. The weighted *R*-factor *wR* and goodness of fit *S* are based on *F*^2^, conventional *R*-factors *R* are based on *F*, with *F* set to zero for negative *F*^2^. The threshold expression of *F*^2^ > σ(*F*^2^) is used only for calculating *R*-factors(gt) *etc*. and is not relevant to the choice of reflections for refinement. *R*-factors based on *F*^2^ are statistically about twice as large as those based on *F*, and *R*- factors based on ALL data will be even larger.

### Fractional atomic coordinates and isotropic or equivalent isotropic displacement parameters (Å^2^)

**Table:**

	*x*	*y*	*z*	*U*_iso_*/*U*_eq_	
O1	−0.07419 (19)	−0.1334 (2)	0.34014 (17)	0.0232 (4)	
O2	−0.0062 (2)	−0.1221 (2)	0.12218 (17)	0.0211 (4)	
O3	0.21206 (18)	0.18228 (18)	−0.05777 (17)	0.0182 (3)	
O4	0.3782 (2)	0.2819 (2)	−0.27523 (17)	0.0208 (4)	
N1	0.2349 (2)	0.0546 (2)	0.1933 (2)	0.0153 (4)	
N2	−0.1227 (2)	0.4135 (2)	0.2298 (2)	0.0195 (4)	
C1	0.4611 (3)	0.1047 (2)	0.0751 (2)	0.0155 (4)	
C2	0.3695 (3)	0.1726 (2)	−0.0437 (2)	0.0170 (5)	
C3	0.4555 (3)	0.2223 (3)	−0.1530 (2)	0.0171 (5)	
C4	0.6201 (3)	0.2052 (3)	−0.1389 (3)	0.0197 (5)	
H4	0.6748	0.2395	−0.2126	0.024*	
C5	0.7084 (3)	0.1396 (3)	−0.0203 (3)	0.0201 (5)	
H5	0.8216	0.1306	−0.0129	0.024*	
C6	0.6296 (3)	0.0881 (3)	0.0856 (3)	0.0189 (5)	
H6	0.6881	0.0413	0.1659	0.023*	
C7	0.3873 (3)	0.0479 (3)	0.1886 (2)	0.0159 (4)	
H7	0.4539	0.0024	0.2653	0.019*	
C8	0.1609 (3)	−0.0021 (3)	0.3116 (2)	0.0155 (4)	
H8	0.2412	−0.0673	0.3675	0.019*	
C9	0.0139 (3)	−0.0931 (3)	0.2577 (2)	0.0163 (5)	
C10	0.1237 (3)	0.1170 (3)	0.4127 (2)	0.0170 (4)	
H10A	0.0851	0.0722	0.4957	0.020*	
H10B	0.2245	0.1686	0.4482	0.020*	
C11	0.0013 (3)	0.2246 (3)	0.3481 (2)	0.0172 (5)	
C12	−0.1594 (3)	0.2345 (3)	0.3819 (2)	0.0158 (4)	
C13	−0.2460 (3)	0.1543 (3)	0.4697 (2)	0.0182 (5)	
H13	−0.1991	0.0732	0.5208	0.022*	
C14	−0.4016 (3)	0.1962 (3)	0.4803 (3)	0.0200 (5)	
H14	−0.4619	0.1427	0.5389	0.024*	
C15	−0.4712 (3)	0.3162 (3)	0.4059 (3)	0.0227 (5)	
H15	−0.5772	0.3437	0.4167	0.027*	
C16	−0.3889 (3)	0.3954 (3)	0.3172 (3)	0.0205 (5)	
H16	−0.4373	0.4754	0.2654	0.025*	
C17	−0.2323 (3)	0.3536 (3)	0.3065 (2)	0.0176 (5)	
C18	0.0167 (3)	0.3350 (3)	0.2562 (2)	0.0189 (5)	
H18	0.1109	0.3548	0.2160	0.023*	
H1N1	0.171 (3)	0.093 (4)	0.108 (3)	0.034 (8)*	
H1N2	−0.141 (3)	0.490 (4)	0.171 (3)	0.024 (7)*	
H1O2	−0.084 (4)	−0.187 (4)	0.102 (3)	0.033 (8)*	
H1O4	0.273 (4)	0.292 (4)	−0.278 (3)	0.046 (10)*	

### Atomic displacement parameters (Å^2^)

**Table:**

	*U*^11^	*U*^22^	*U*^33^	*U*^12^	*U*^13^	*U*^23^
O1	0.0195 (8)	0.0321 (10)	0.0189 (8)	−0.0065 (7)	0.0056 (6)	0.0020 (8)
O2	0.0196 (8)	0.0255 (10)	0.0191 (8)	−0.0075 (7)	0.0058 (6)	−0.0043 (7)
O3	0.0124 (7)	0.0216 (8)	0.0210 (8)	0.0019 (6)	0.0043 (6)	0.0020 (7)
O4	0.0162 (8)	0.0266 (9)	0.0208 (8)	0.0010 (7)	0.0069 (7)	0.0035 (7)
N1	0.0142 (9)	0.0166 (9)	0.0157 (9)	0.0013 (7)	0.0039 (7)	0.0001 (8)
N2	0.0194 (10)	0.0179 (10)	0.0228 (10)	0.0010 (8)	0.0087 (8)	0.0028 (9)
C1	0.0137 (10)	0.0168 (11)	0.0166 (10)	−0.0014 (8)	0.0039 (8)	−0.0031 (9)
C2	0.0143 (10)	0.0162 (11)	0.0208 (11)	0.0007 (8)	0.0041 (8)	−0.0019 (9)
C3	0.0169 (10)	0.0152 (11)	0.0197 (11)	−0.0008 (9)	0.0047 (8)	−0.0017 (9)
C4	0.0170 (11)	0.0221 (13)	0.0218 (11)	−0.0026 (9)	0.0086 (9)	−0.0004 (10)
C5	0.0121 (10)	0.0234 (12)	0.0250 (11)	−0.0021 (9)	0.0037 (9)	−0.0021 (10)
C6	0.0146 (10)	0.0225 (12)	0.0191 (11)	−0.0006 (9)	0.0012 (8)	0.0003 (10)
C7	0.0159 (10)	0.0144 (10)	0.0169 (10)	−0.0006 (8)	0.0006 (8)	−0.0024 (9)
C8	0.0136 (10)	0.0182 (11)	0.0149 (10)	0.0002 (9)	0.0028 (8)	0.0010 (9)
C9	0.0122 (10)	0.0162 (11)	0.0203 (11)	0.0004 (8)	0.0022 (8)	0.0021 (9)
C10	0.0150 (10)	0.0200 (12)	0.0165 (10)	0.0012 (9)	0.0040 (8)	−0.0001 (9)
C11	0.0166 (10)	0.0182 (11)	0.0170 (10)	−0.0010 (9)	0.0032 (8)	−0.0026 (9)
C12	0.0150 (10)	0.0177 (11)	0.0152 (10)	−0.0004 (9)	0.0043 (8)	−0.0036 (9)
C13	0.0195 (11)	0.0182 (12)	0.0171 (10)	0.0007 (9)	0.0038 (8)	−0.0011 (9)
C14	0.0181 (11)	0.0202 (12)	0.0230 (11)	−0.0031 (9)	0.0080 (9)	−0.0011 (10)
C15	0.0166 (11)	0.0228 (13)	0.0300 (13)	−0.0003 (9)	0.0074 (9)	−0.0049 (11)
C16	0.0188 (11)	0.0161 (11)	0.0272 (12)	0.0033 (8)	0.0054 (9)	−0.0002 (10)
C17	0.0182 (11)	0.0168 (11)	0.0189 (11)	−0.0017 (9)	0.0065 (8)	−0.0021 (9)
C18	0.0166 (11)	0.0207 (12)	0.0200 (11)	−0.0007 (9)	0.0048 (9)	−0.0036 (10)

### Geometric parameters (Å, °)

**Table:**

O1—C9	1.218 (3)	C6—H6	0.9500
O2—C9	1.301 (3)	C7—H7	0.9500
O2—H1O2	0.89 (3)	C8—C9	1.525 (3)
O3—C2	1.318 (3)	C8—C10	1.530 (3)
O4—C3	1.361 (3)	C8—H8	1.0000
O4—H1O4	0.89 (3)	C10—C11	1.501 (3)
N1—C7	1.295 (3)	C10—H10A	0.9900
N1—C8	1.465 (3)	C10—H10B	0.9900
N1—H1N1	0.97 (3)	C11—C18	1.367 (4)
N2—C18	1.375 (3)	C11—C12	1.442 (3)
N2—C17	1.378 (3)	C12—C13	1.405 (3)
N2—H1N2	0.90 (3)	C12—C17	1.409 (3)
C1—C2	1.417 (3)	C13—C14	1.387 (3)
C1—C6	1.419 (3)	C13—H13	0.9500
C1—C7	1.426 (3)	C14—C15	1.403 (4)
C2—C3	1.429 (3)	C14—H14	0.9500
C3—C4	1.384 (3)	C15—C16	1.382 (4)
C4—C5	1.395 (3)	C15—H15	0.9500
C4—H4	0.9500	C16—C17	1.396 (3)
C5—C6	1.373 (3)	C16—H16	0.9500
C5—H5	0.9500	C18—H18	0.9500
			
C9—O2—H1O2	110 (2)	C10—C8—H8	107.0
C3—O4—H1O4	115 (2)	O1—C9—O2	124.9 (2)
C7—N1—C8	123.31 (19)	O1—C9—C8	119.9 (2)
C7—N1—H1N1	115.0 (18)	O2—C9—C8	115.22 (19)
C8—N1—H1N1	121.5 (18)	C11—C10—C8	114.84 (18)
C18—N2—C17	108.2 (2)	C11—C10—H10A	108.6
C18—N2—H1N2	126.3 (17)	C8—C10—H10A	108.6
C17—N2—H1N2	125.5 (17)	C11—C10—H10B	108.6
C2—C1—C6	121.6 (2)	C8—C10—H10B	108.6
C2—C1—C7	121.40 (19)	H10A—C10—H10B	107.5
C6—C1—C7	117.0 (2)	C18—C11—C12	106.1 (2)
O3—C2—C1	122.1 (2)	C18—C11—C10	129.5 (2)
O3—C2—C3	121.2 (2)	C12—C11—C10	124.3 (2)
C1—C2—C3	116.62 (19)	C13—C12—C17	119.6 (2)
O4—C3—C4	118.2 (2)	C13—C12—C11	133.6 (2)
O4—C3—C2	121.44 (19)	C17—C12—C11	106.9 (2)
C4—C3—C2	120.3 (2)	C14—C13—C12	118.5 (2)
C3—C4—C5	122.3 (2)	C14—C13—H13	120.8
C3—C4—H4	118.9	C12—C13—H13	120.8
C5—C4—H4	118.9	C13—C14—C15	121.1 (2)
C6—C5—C4	119.1 (2)	C13—C14—H14	119.5
C6—C5—H5	120.4	C15—C14—H14	119.5
C4—C5—H5	120.4	C16—C15—C14	121.4 (2)
C5—C6—C1	120.1 (2)	C16—C15—H15	119.3
C5—C6—H6	119.9	C14—C15—H15	119.3
C1—C6—H6	119.9	C15—C16—C17	117.7 (2)
N1—C7—C1	123.8 (2)	C15—C16—H16	121.2
N1—C7—H7	118.1	C17—C16—H16	121.2
C1—C7—H7	118.1	N2—C17—C16	130.1 (2)
N1—C8—C9	111.15 (18)	N2—C17—C12	108.1 (2)
N1—C8—C10	111.87 (19)	C16—C17—C12	121.8 (2)
C9—C8—C10	112.33 (18)	C11—C18—N2	110.8 (2)
N1—C8—H8	107.0	C11—C18—H18	124.6
C9—C8—H8	107.0	N2—C18—H18	124.6
			
C6—C1—C2—O3	176.6 (2)	C9—C8—C10—C11	−61.5 (3)
C7—C1—C2—O3	−2.4 (3)	C8—C10—C11—C18	−75.8 (3)
C6—C1—C2—C3	0.5 (3)	C8—C10—C11—C12	108.5 (3)
C7—C1—C2—C3	−178.4 (2)	C18—C11—C12—C13	−179.4 (2)
O3—C2—C3—O4	0.3 (3)	C10—C11—C12—C13	−2.8 (4)
C1—C2—C3—O4	176.4 (2)	C18—C11—C12—C17	−0.2 (2)
O3—C2—C3—C4	−177.1 (2)	C10—C11—C12—C17	176.4 (2)
C1—C2—C3—C4	−1.0 (3)	C17—C12—C13—C14	−0.5 (3)
O4—C3—C4—C5	−177.2 (2)	C11—C12—C13—C14	178.6 (2)
C2—C3—C4—C5	0.3 (4)	C12—C13—C14—C15	−0.3 (3)
C3—C4—C5—C6	0.9 (4)	C13—C14—C15—C16	1.3 (4)
C4—C5—C6—C1	−1.3 (4)	C14—C15—C16—C17	−1.3 (4)
C2—C1—C6—C5	0.6 (4)	C18—N2—C17—C16	178.8 (2)
C7—C1—C6—C5	179.6 (2)	C18—N2—C17—C12	−0.3 (3)
C8—N1—C7—C1	−179.0 (2)	C15—C16—C17—N2	−178.5 (2)
C2—C1—C7—N1	−0.7 (4)	C15—C16—C17—C12	0.5 (4)
C6—C1—C7—N1	−179.7 (2)	C13—C12—C17—N2	179.6 (2)
C7—N1—C8—C9	−132.3 (2)	C11—C12—C17—N2	0.3 (2)
C7—N1—C8—C10	101.2 (2)	C13—C12—C17—C16	0.4 (3)
N1—C8—C9—O1	−170.5 (2)	C11—C12—C17—C16	−178.9 (2)
C10—C8—C9—O1	−44.3 (3)	C12—C11—C18—N2	0.0 (3)
N1—C8—C9—O2	9.7 (3)	C10—C11—C18—N2	−176.3 (2)
C10—C8—C9—O2	135.9 (2)	C17—N2—C18—C11	0.2 (3)
N1—C8—C10—C11	64.4 (2)		

### Hydrogen-bond geometry (Å, °)

**Table:**

*D*—H···*A*	*D*—H	H···*A*	*D*···*A*	*D*—H···*A*
N1—H1N1···O3	0.97 (3)	1.86 (3)	2.646 (2)	136 (2)
N2—H1N2···O3^i^	0.90 (3)	2.13 (3)	3.020 (2)	170 (2)
O2—H1O2···O3^ii^	0.89 (3)	1.64 (4)	2.526 (2)	174 (4)
O4—H1O4···O3	0.89 (3)	2.45 (3)	2.823 (2)	106 (2)
O4—H1O4···O1^i^	0.89 (3)	1.83 (3)	2.665 (2)	156 (3)
C7—H7···O4^iii^	0.95	2.49	3.197 (3)	132

Symmetry codes: (i) −*x*, *y*+1/2, −*z*; (ii) −*x*, *y*−1/2, −*z*; (iii) −*x*+1, *y*−1/2, −*z*.
